# Missed Monteggia fractures in children treated by open reduction of the radial head and corrective osteotomy of the ulna

**DOI:** 10.1038/s41598-022-21019-4

**Published:** 2022-10-07

**Authors:** Shu Cao, Zhong-Gen Dong, Li-Hong Liu, Jian-Wei Wei, Zhao-Biao Luo, Ping Peng

**Affiliations:** 1 Department of Orthopedics, The Second Xiangya Hospital, Central South University, No. 139 Renmin Road, Changsha, 410011 Hunan People’s Republic of China; 2 Department of Orthopedics, Hunan Provincial People’s Hospital (The First Affiliated Hospital of Hunan Normal University), Changsha, 410005 Hunan People’s Republic of China

**Keywords:** Bone, Bone quality and biomechanics

## Abstract

Missed Monteggia fractures in children may cause pain, deformity, decreased range of motion, neurological symptoms, and late arthritis of the elbow. Numerous surgical techniques have been advocated to reconstruct missed Monteggia lesions. The purpose of the present study were first to evaluate the clinical and radiographic outcomes after open reduction of the radial head and corrective osteotomy of the ulna, second to identify the factors associated with the preoperative radial notch/head appearance and the postoperative radiographic results. This study investigated the preoperative MRI presentation and the treatment of 29 patients who were diagnosed missed Monteggia fracture. Radiologic and clinical results of these patients were evaluated retrospectively, and the patient’s and surgical factors related to preoperative radial notch/head appearance and the postoperative radiographic results were analyzed. Of the 29 patients, the average Kim elbow performance score at the last follow-up was 93.6, with 25 excellent, three good, one fair, and no poor results. 19 children had reduced radial heads, 8 had a subluxated radial head and 2 had dislocated radial heads at the last follow-up. The patient’s gender and age had no significant influence on the appearance of radial notch/head and final radiographic results. However, the appearance of radial notch/head can significantly affect the final radiographic result (P < 0.001). The interval time was an important factor which related with the appearance of radial notch/head and final radiographic results (P < 0.001). Treating a missed Monteggia fracture by open reduction of the radial head and corrective osteotomy of the ulna is generally successful and preoperative MRI is meaningful for evaluation of the condition of the radial head and the radial notch which is related with the final radiographic result. The interval time from injury to operation exceeds 6 months, the risk of radial notch/head abnormality and radial head subluxation/re-dislocation after operation significantly increase.

## Introduction

Monteggia lesions represent 1% of all forearm fractures^1^, and acute Monteggia lesions are sometimes neglected because of the non-standard radiograph, plastic deformation of the ulna and lack of physician experience^2,3^. Boyd and Boals defined missed Monteggia lesions as at least 4 weeks after injury^4^. In missed Monteggia lesions, due to the lack of joint restraint and concavity, the radial head and the radial notch of the proximal ulna will overgrow^5,6^, thus leading to unacceptable long-term results for the patient^7^, including pain, deformity, dysfunction and late arthritis of the elbow^8,9^. Hence, treatment for chronic radial head dislocation is indicated at the earliest time to restore the normal anatomy^10^. A series of surgical options have been described in the literatures, including open reduction of the radial head^11,12^ and ulnar osteotomy with or without reconstruction of the annular ligament^13–18^; indirect reduction of the radial head by overcorrection the ulna^13^; non-operative treatment and radial head resection at skeletal maturity^14^. Some authors have suggested that patients who are more than 12 years old and the interval time between injury and operation is more than 3 years, reduction of the radial head is not recommended^15–18^. For the preoperative treatment plan, the age of the patient and the time interval are key factors that affect the surgical outcome, but little attention has been paid to the morphologic features of the proximal radioulnar joint, which is considered quite important for reducing the radial head to the correct position^19,20^. However, only X-ray and computerized tomography (CT) ^5,6^ have been described as preoperative the proximal radioulnar joint studies, but the soft tissue or cartilaginous may not be well assessed on X-ray and CT. Little attention has been given to magnetic resonance imaging (MRI) of the abnormal elbow and interosseous membrane^21^.

At our hospital, we have performed both X-ray and MRI preoperatively to evaluated the pathologic changes of the elbow, then managed patients with a missed Monteggia lesion by open reduction of the radial head and ulnar osteotomy, with reconstruction of the annular ligament and transcapitellar K-wire in radial head instability cases. In this study, our aim were first to evaluate the clinical and radiographic outcomes after open reduction of the radial head and corrective osteotomy of the ulna, second to identify the factors associated with the preoperative radial notch/head appearance evaluation and the postoperative radiographic results.

## Results

All patients underwent open reduction of the radial head and ulna osteotomy, and 12 had concurrent reconstruction of the annular ligament and a humeroradial pin. Two patients (cases 2 and 3) had a posterior interosseous nerve palsy preoperatively. Nerve palsies recovered spontaneously 6 months after surgery. One patient (case 4) had an associated humeral lateral condyle fracture, and one (case 3) had a concurrent distal radius metaphysis fracture. Two patients (cases 4 and 11) had heterotopic ossification at first presentation, and heterotopic ossification resolved spontaneously 1 year after surgery. Patient demographics, the preoperative MRI presentation, the surgical procedure undertaken, and the radiographic results are shown in Table 1.

**Table 1:** Demographic data of the 29 children.

Case	Sex	Side	Age (years)	Preoperative delay (months)	MRI presentation		Treatment	Final radiographic result (radial head coverage)
					Radial head	Radial notch		
1	F	L	10	1	Concave	Concave	OR + UO	Concentric (Grade 1)
2	M	R	7	8	Concave	Domed	OR + UO + ALR + HR	subluxated (Grade 3)
3	F	L	4	1	Concave	Concave	OR + UO + ALR + HR	Concentric (Grade 1)
4	F	R	9	1	Concave	Concave	OR + UO	Concentric (Grade 1)
5	M	L	6	2	Concave	Concave	OR + UO	Concentric (Grade 1)
6	F	L	5	2	Concave	Concave	OR + UO	Concentric (Grade 1)
7	M	L	7	48	Domed	Domed	OR + UO + ALR + HR	Subluxated (Grade 4)
8	M	L	7	6	Concave	Concave	OR + UO	Concentric (Grade 1)
9	F	L	5	10	Concave	Flat	OR + UO	Concentric (Grade 1)
10	M	L	6	12	Concave	Domed	OR + UO	Subluxated (Grade 2)
11	M	L	8	2	Concave	Concave	OR + UO	Concentric (Grade 1)
12	M	L	13	84	Domed	Domed	OR + UO + ALR + HR	Dislocated (Grade 5)
13	F	R	5	3	Concave	Concave	OR + UO	Concentric (Grade 1)
14	M	L	6	1	Concave	Concave	OR + UO	Concentric (Grade 1)
15	F	L	4	2	Concave	Concave	OR + UO + ALR + HR	Concentric (Grade 1)
16	M	R	8	2	Concave	Concave	OR + UO	Concentric (Grade 1)
17	F	R	5	1	Concave	Concave	OR + UO + ALR + HR	Concentric (Grade 1)
18	M	L	9	60	Flat	Domed	OR + UO + ALR + HR	Subluxated (Grade 3)
19	M	R	5	1	Concave	Concave	OR + UO	Concentric (Grade 1)
20	F	L	5	10	Concave	Flat	OR + UO	Concentric (Grade 1)
21	M	R	8	1	Concave	Concave	OR + UO	Concentric (Grade 1)
22	M	L	7	24	Domed	Domed	OR + UO + ALR + HR	Subluxated (Grade 4)
23	M	L	6	1	Concave	Concave	OR + UO	Concentric (Grade 1)
24	M	R	3	12	Flat	Domed	OR + UO + ALR + HR	Subluxated (Grade 3)
25	M	L	7	1	Concave	Concave	OR + UO + ALR + HR	Concentric (Grade 1)
26	M	R	12	3	Concave	Concave	OR + UO	Subluxated (Grade 3)
27	M	R	11	10	Domed	Domed	OR + UO	Subluxated (Grade 3)
28	F	R	8	1	Concave	Concave	OR + UO	Dislocated (Grade 5)
29	M	L	12	2	Concave	Concave	OR + UO + ALR + HR	Concentric (Grade 1)

*UO* ulnar osteotomy, *OR* open reduction of radial head, *ALR* annular ligament reconstruction, *HR* humeroradial pin.

### Clinical results

The average Kim elbow performance score at the last follow-up was 93.6, with 25 excellent, three good, one fair, and no poor results. One patient (case 12) complained of intermittent mild pain that did not cause limitations in daily life. No patient had active elbow extension limitations before or after treatment. Compared with the preoperative range of movement, there was no significant difference in postoperative forearm pronation (P = 0.82) and supination (P = 0.07). Elbow flexion and Kim score improved significantly (P < 0.05) (Table 2).

**Table 2:** Pre- and postoperative range of movement and Kim elbow score ($\tilde{x}$ ± *s*).

	Pronation (°) (mean ± SD)	Supination (°) (mean ± SD)	Flexion (°) (mean ± SD)	Kim score (mean ± SD)
preoperative	67.8 ± 5.6	67.9 ± 6.1	111.7 ± 8.0	88.4 ± 10.8
postoperative	67.9 ± 4.7	69.8 ± 6.3	123.4 ± 16.3	93.6 ± 9.7
*t*	− 0.23	− 1.89	− 4.63	− 4.85
*P* value*	0.82	0.07	0.00	0.00

*t test; *P* ≤ 0.05, statistically significant.

### Radiological results

**Figure 1:**
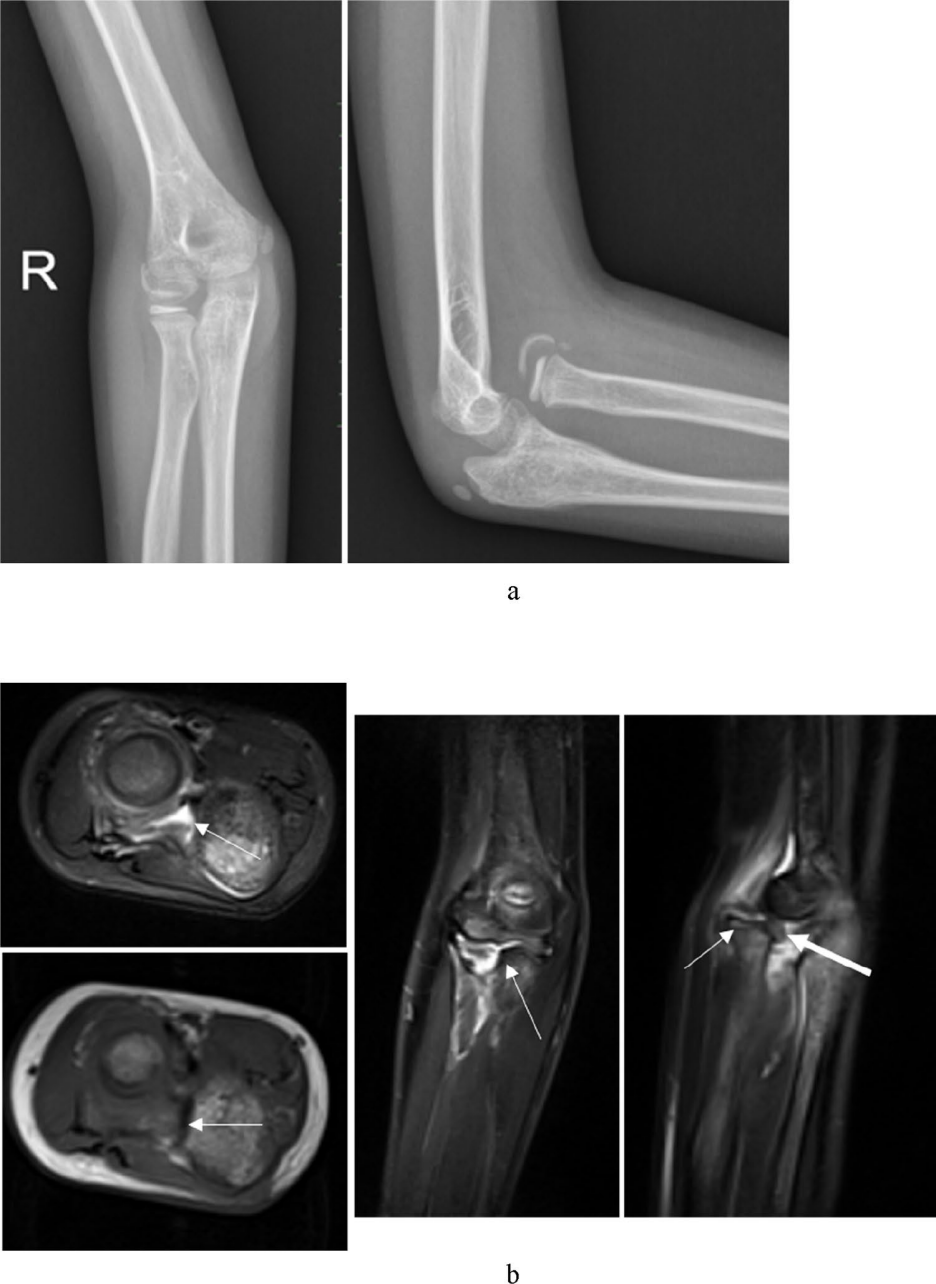

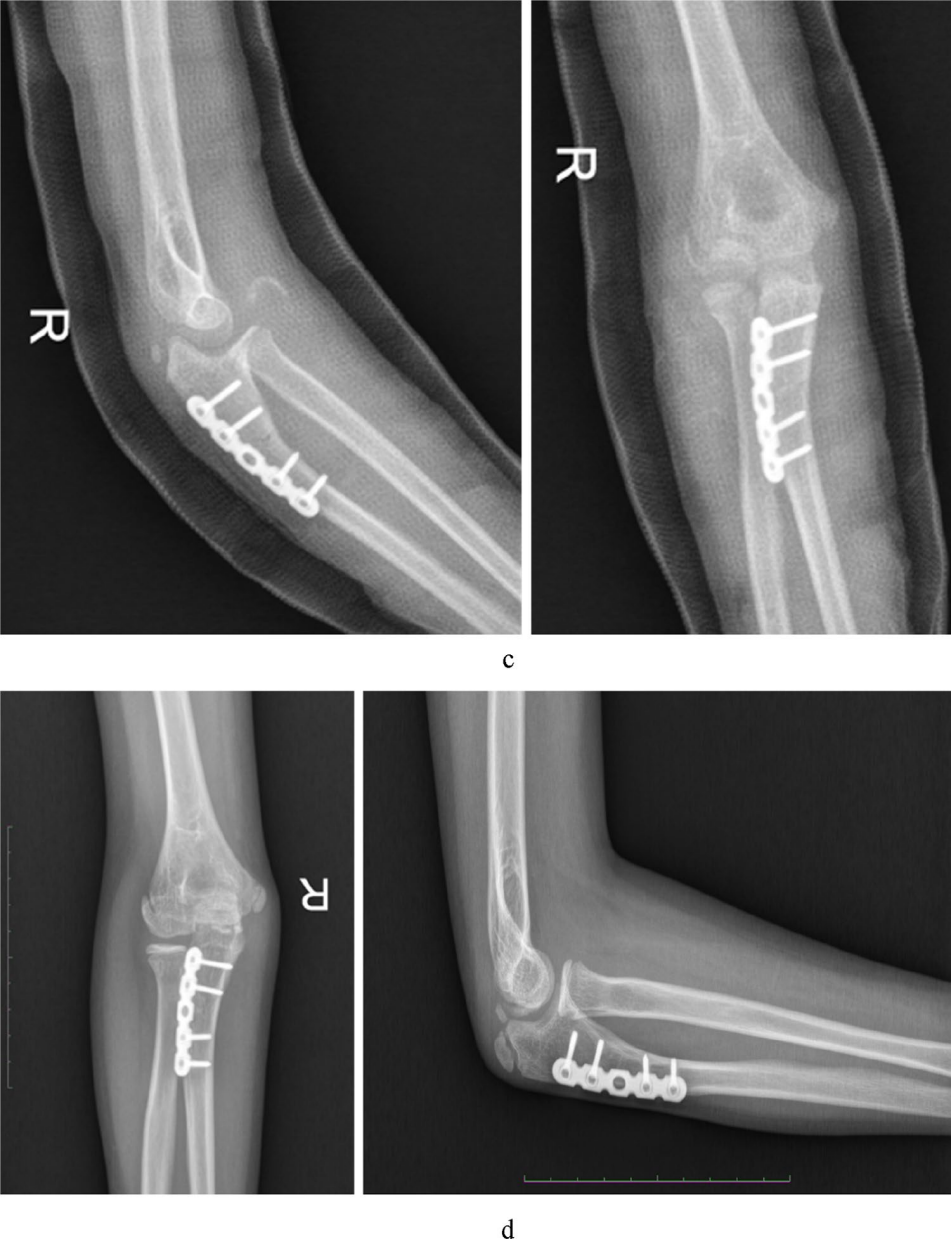
A 9 years old girl (case 4) had fallen from chair. She underwent treatment for a chronic Monteggia fracture after 1 month. (**a**) Preoperative X-ray. Heterotopic ossification can be seen. (**b**) Preoperative MRI. Axial T1- and T2-weighted images demonstrated the dislocation of the proximal radioulnar joint; axial and coronal T2-weighted images showed the concave radial notch of the ulna (the arrow); sagittal T2-weighted images displayed the dislocation of the humeroradial joint and the concave radial head (the thin arrow) combined with the folded and tortuousness annual ligament (the gross arrow). (**c**) Open reduction with ulnar osteotomy was undertaken. (**d**) The radial head remained concentric (Grade 1) and heterotopic ossification disappeared at 1 years after operation.

**Figure 2:**
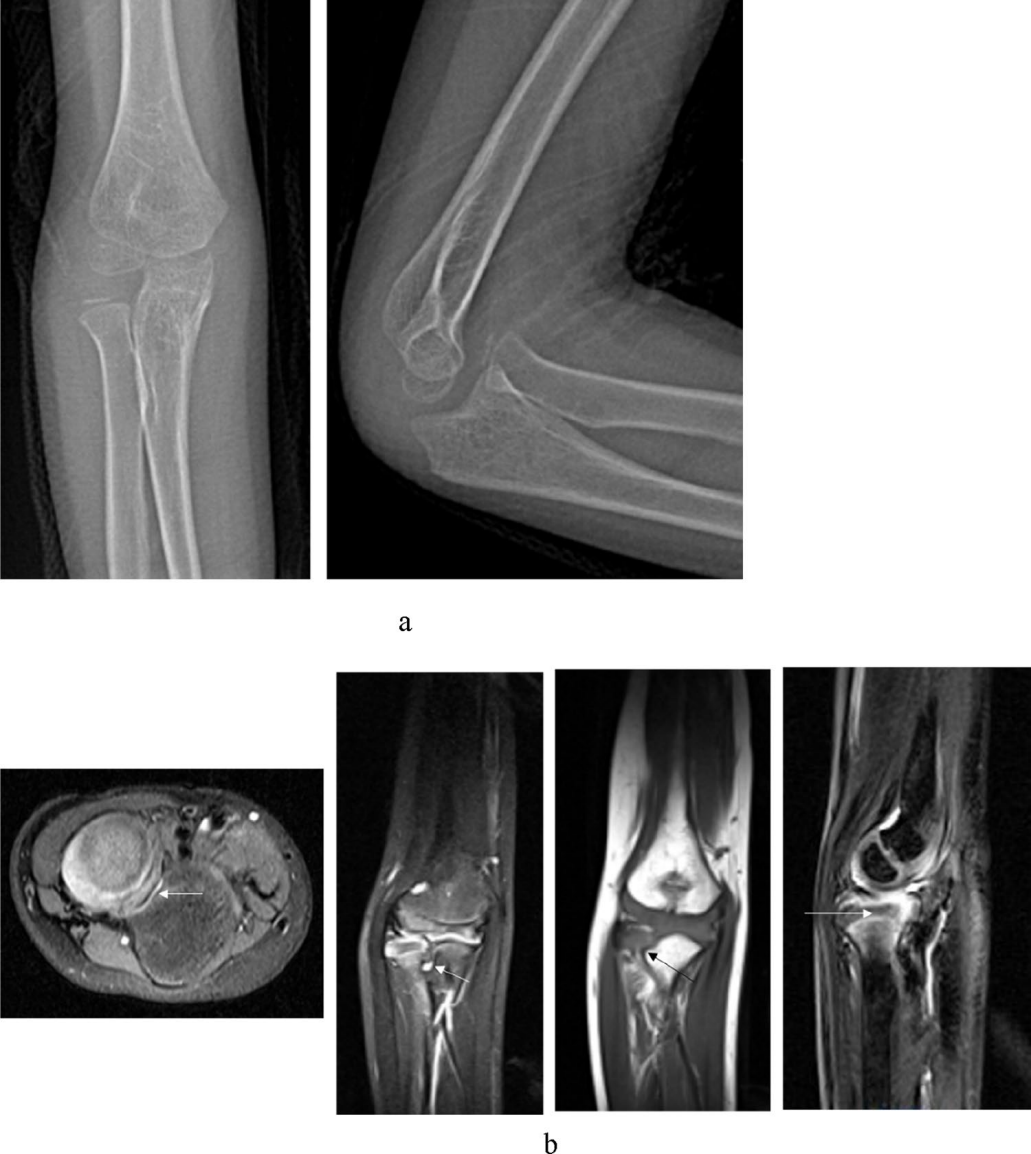

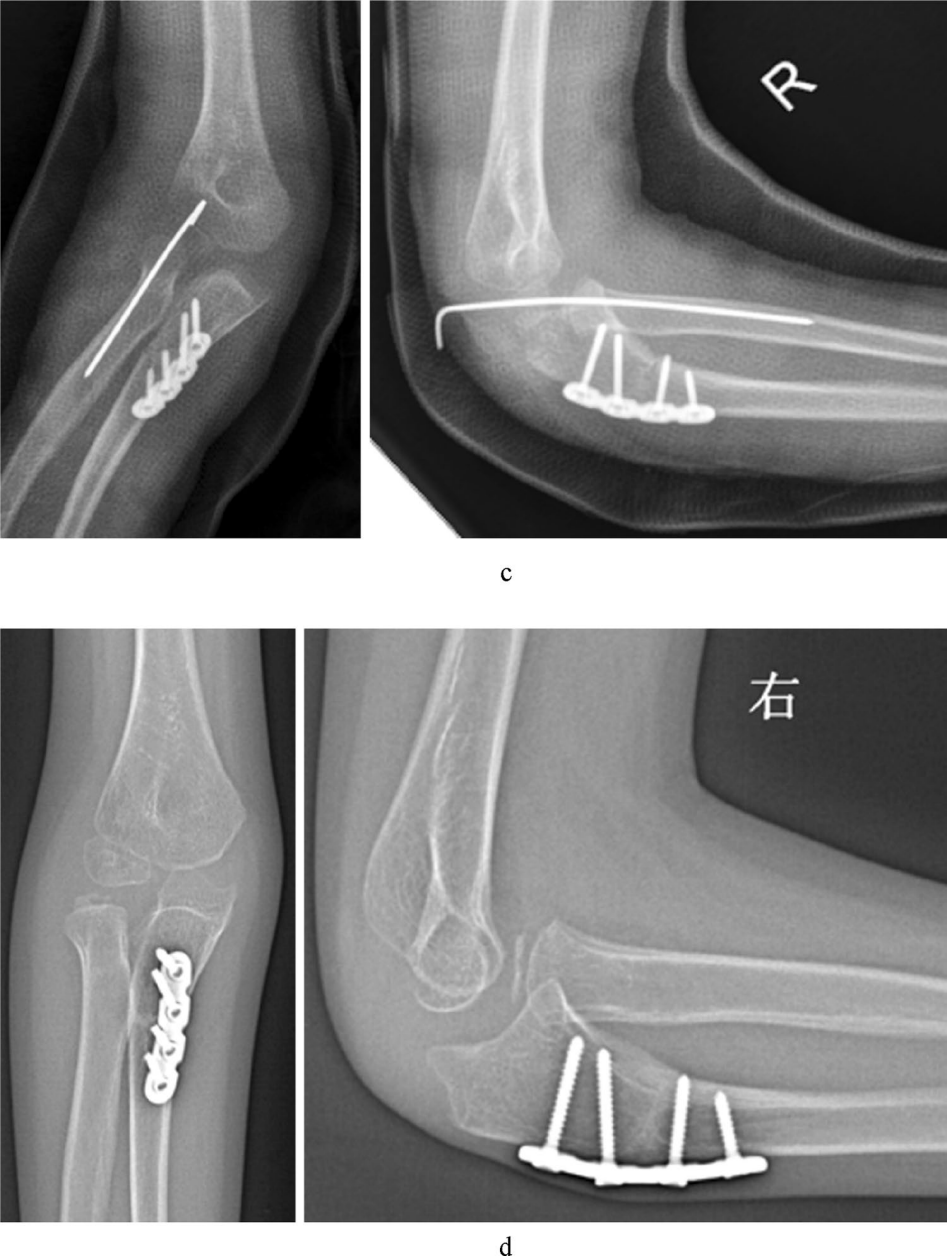
Imaging of a 7 years old boy (case 2) referred for the treatment of missed Monteggia lesion. His internal time between injury and operation was 8 months. (**a**) Preoperative X-ray. (**b**) Preoperative MRI. Axial T2-weighted images demonstrated the proximal radioulnar joint was dislocated and the appearance of the radial notch (the arrow) was flat; coronal T1- and T2-weighted images showed the radial notch of the ulna was hyperplasia (the arrow); sagittal T1-weighted images showed the dislocation of the humeroradial joint and the concave radial head (the arrow) combined with the folded and tortuousness annual ligament in the joint. (**c**) Open reduction and ulnar osteotomy with reconstruction of the annular ligament and a humeroradial pin was undertaken. (**d**) The radial head subluxated (Grade 3) at 2 years after operation.

**Figure 3:**
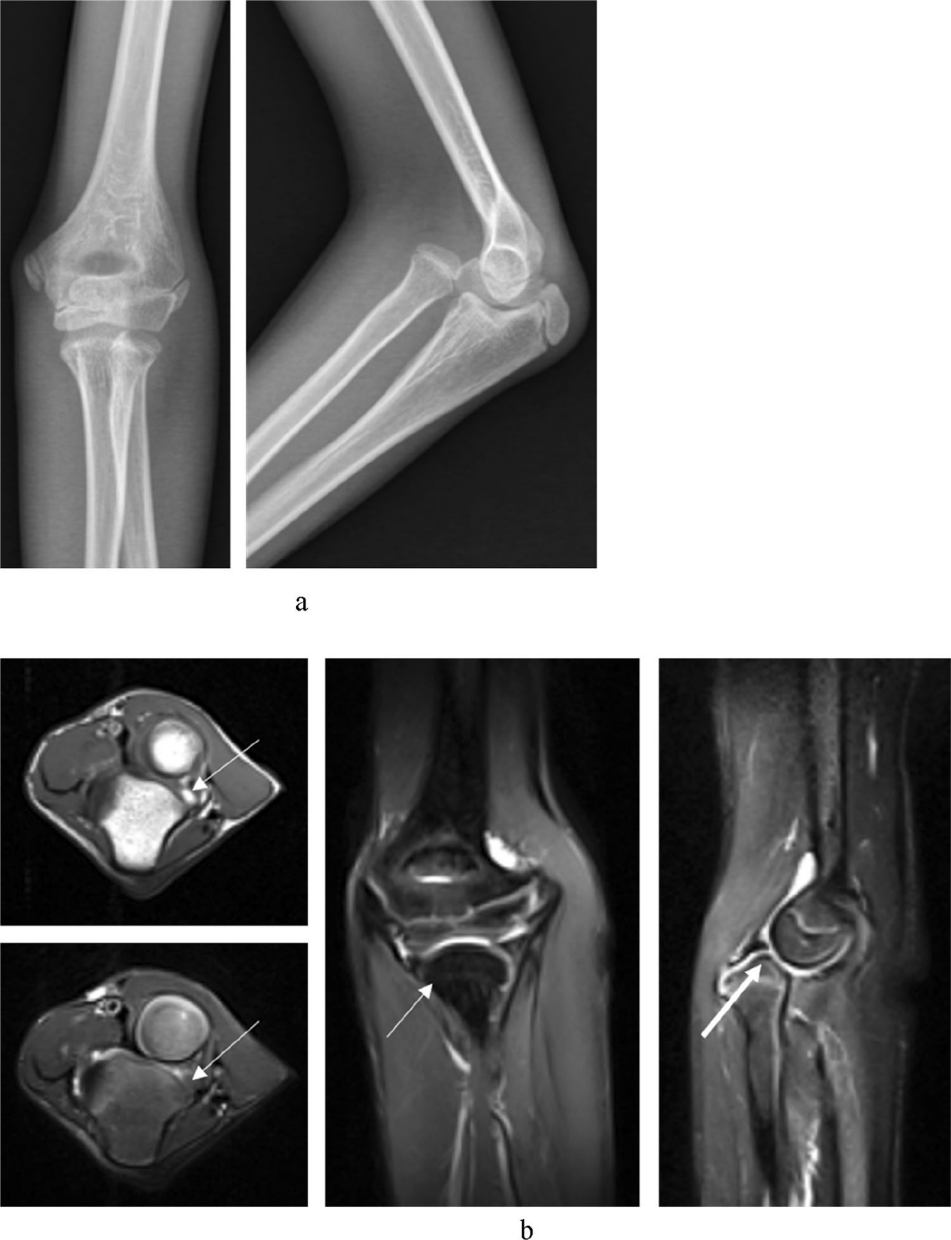

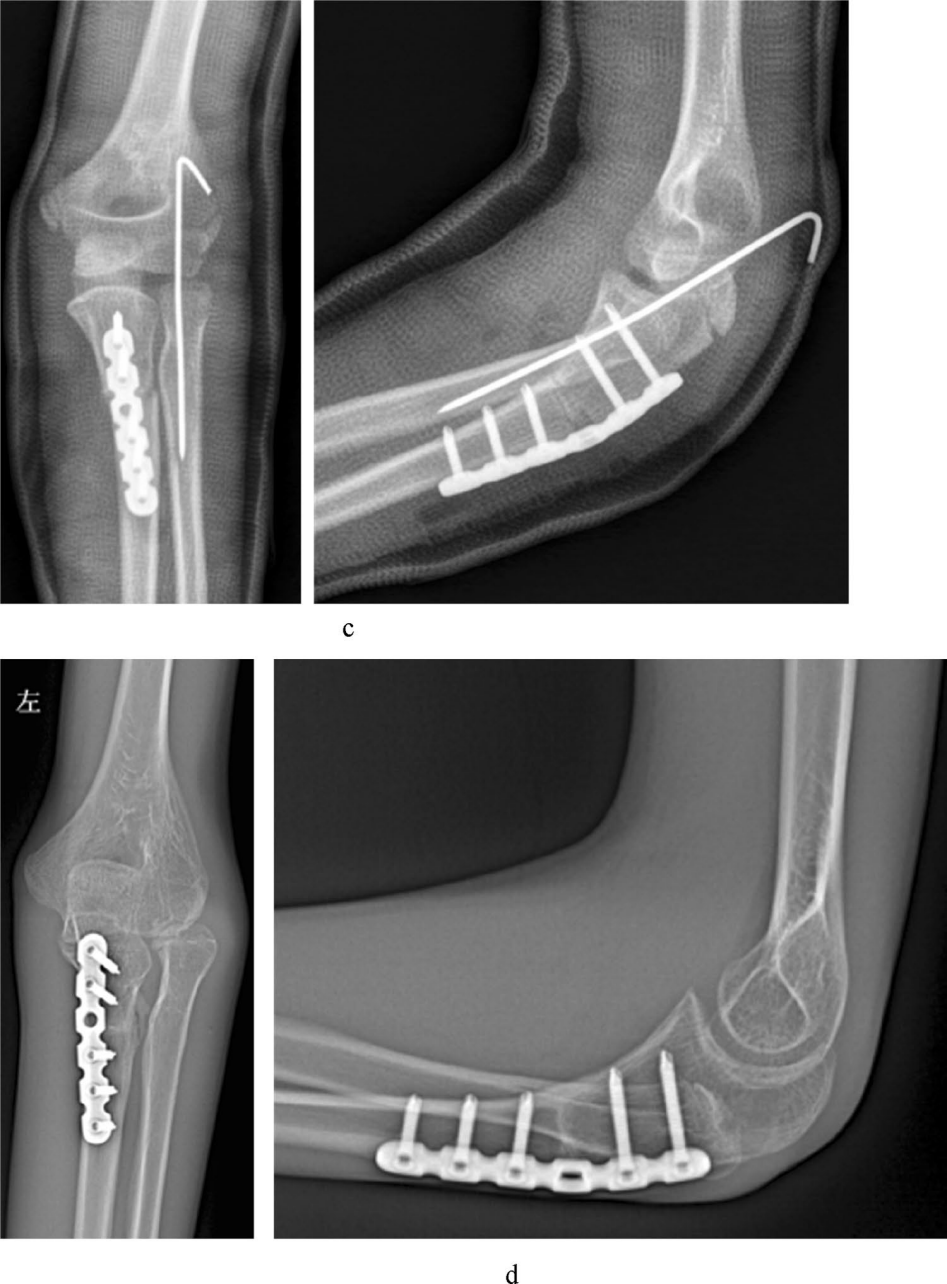
A 13 years old boy (case 12) with a missed Monteggia lesion after a 7 years interval. (**a**) Preoperative X-ray. The appearance of the radial head was domed. (**b**) Preoperative MRI. Axial T1- and T2-weighted images showed the proximal radioulnar joint was dislocated and the appearance of the radial notch (the arrow) was domed; coronal T2-weighted images showed the radial notch of the ulna was severe hyperplasia (the arrow); sagittal T2-weighted images showed the dislocation of the humeroradial joint and the domed radial head (the arrow) with dense fibrous scar tissue. (**c**) Open reduction and ulnar osteotomy with reconstruction of the annular ligament and a humeroradial pin was undertaken. (**d**) The radial head dislocated (Grade 5) due to the hyperplastic radial head incongruency with the capitellum and the elbow had osteoarthritic changes posteriorly at 3 years after operation.

**Figure 4:**
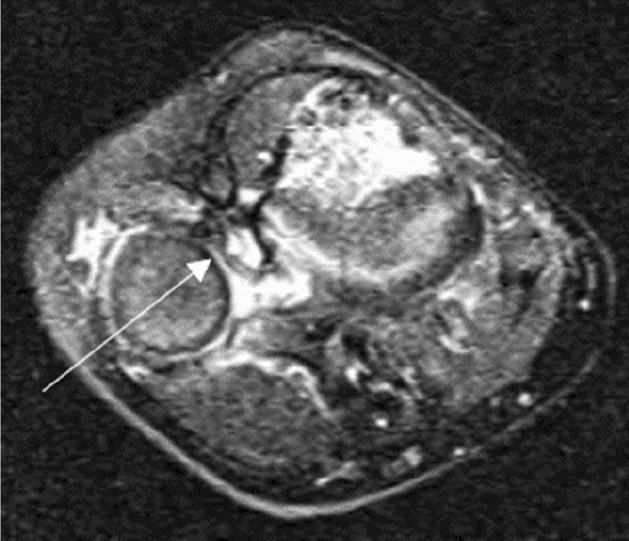
T2-weighted images displayed obvious tear of the annular ligament (the arrow) of the case 14.

**Figure 5:**
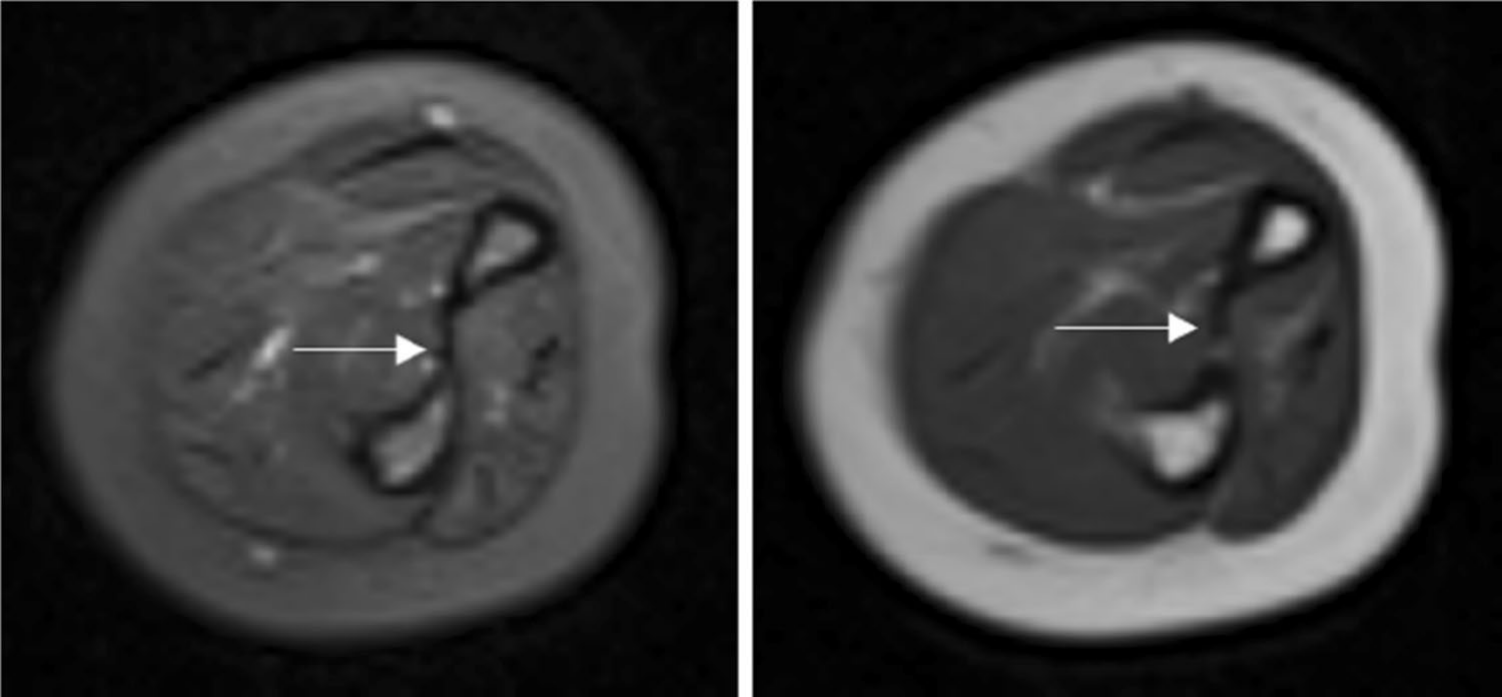
Axial T1 and T2-weighted images of the forearm (case 10) demonstrated no obvious the injuries of the interosseous membrane (the arrow).

**Figure 6:**
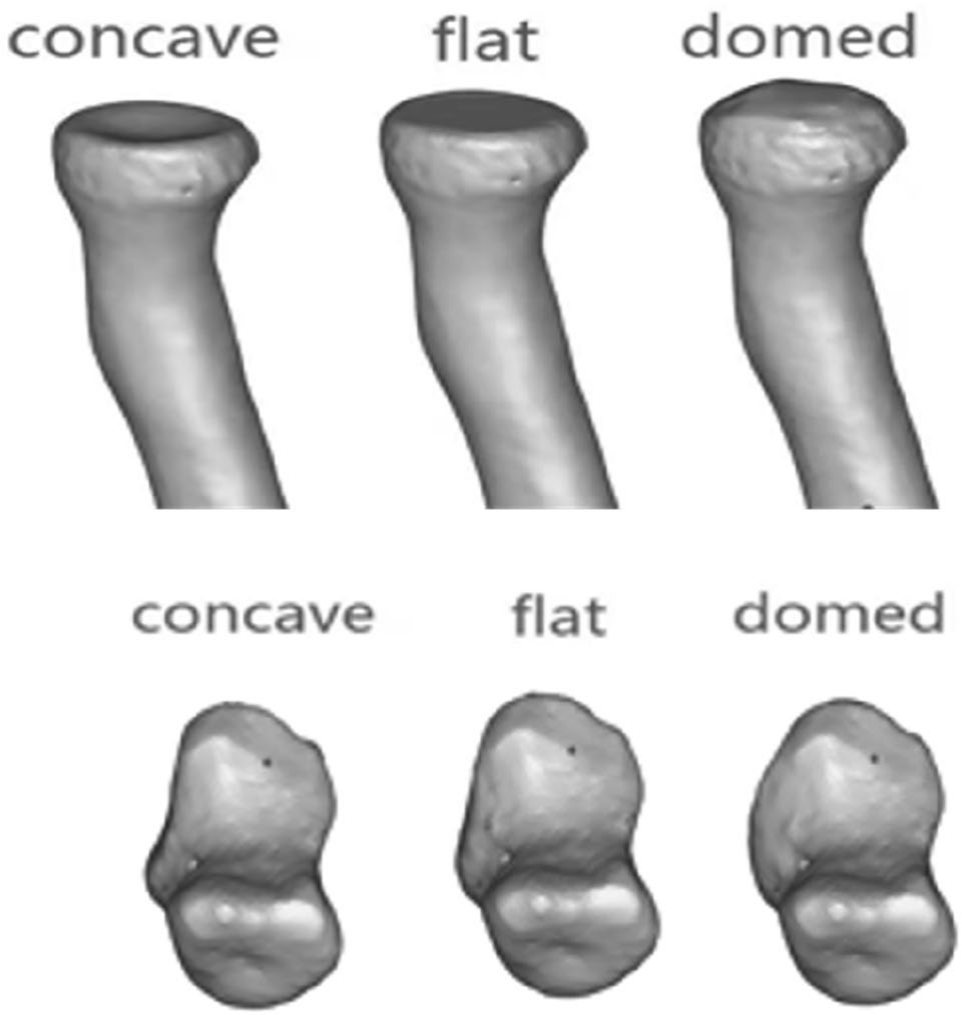
The patterns of the morphologic features of the radial head and radial notch, concave, flat, and domed, are shown.

From preoperative MRI, all the patients had radial head dislocation and tortuosity of the annular ligament (Fig. 1b), 10 patients had deformities of the radial notch of the ulna (Figs. 2b, 3b), 6 patients had deformities of the radial head (Fig. 3b), and one patient had obvious tears of the annular ligament (Fig. 4). No patients had obvious injuries of the interosseous membrane (Fig. 5).

Postoperative radiological assessment used the last standard anteroposterior and lateral radiographs of the elbow. Overall, 19 children had reduced radial heads, 8 had a subluxated radial head (Fig. 2), and two had dislocated radial heads (Fig. 3).

Univariate analysis was carried out in an attempt to identify factors associated with the preoperative radial notch/head appearance and the postoperative radiographic results. These factors included age > 7 years, gender, neglected for more than 6 months and the surgical procedure were evaluated with the preoperative radial notch/head appearance. These factors included age > 7 years, gender, neglected for more than 6 months, the appearance of the radial notch and radial head, the surgical procedure were evaluated with the postoperative radiographic results.

No significant differences were found in age, gender and surgical procedure correlated with the preoperative radial notch/head appearance (Table 3). The rate of radial notch/head abnormality in the group of interval time more than 6 months was higher than that in the group with interval time ≤ 6 months, the difference was statistically significant (P < 0.001).

**Table 3:** Univariate analysis of factors that might affect the preoperative radial notch/head appearance.

Parameters	Concave radial notch/head (n = 19)	Flat/domed radial notch/head (n = 10)	P value*
**Age (years)**			1.00
≤ 7	12 (66.67)	6 (33.33)	
> 7	7 (63.64)	4 (36.36)	
**Gender**			0.43
Male	12 (60.00)	8 (40.00)	
Female	7 (77.78)	2 (22.22)	
**Interval time (months)**			
≤ 6	19 (100)	0	< 0.001
> 6	0	10 (100)	
**Surgical procedure**			
UO + OR	13 (76.47)	4 (23.53)	0.24
UO + OR + ALR + HR	6 (50.00)	6 (50.00)	

*Fisher’s exact test; *P* ≤ 0.05, statistically significant.

No significant differences were found in age, gender and operation ways correlated with the postoperative radiographic results (Table 4). The rate of radial head subluxation/dislocation in the group of interval time more than 6 months was higher than that in the group with interval time ≤ 6 months, the difference was statistically significant (P < 0.001). The concave radial notch/head group had a lower rate of radial head subluxation/dislocation than the flat/domed radial notch/head group, the difference was statistically significant (P < 0.001).

**Table 4:** Univariate analysis of factors that might affect the postoperative radiographic results.

Parameters	Concentric radial head (n = 19)	Subluxated/dislocated radial head (n = 10)	P value*
**Age (years)**			0.43
≤ 7	13 (72.22)	5 (27.78)	
> 7	6 (54.55)	5 (45.45)	
**Gender**			0.11
Male	11 (55.00)	9 (45.00)	
Female	8 (88.89)	1 (11.11)	
**Interval time (months)**			
≤ 6	17 (89.47)	2 (10.53)	< 0.001
> 6	2 (20.00)	8 (80.00)	
**Appearance of radial notch/head**			< 0.001
Concave	17 (94.44)	1 (5.56)	
Flat/domed	2 (18.19)	9 (81.82)	
**Surgical procedure**			0.24
UO + OR	13 (76.47)	4 (23.53)	
UO + OR + ALR + HR	6 (50.00)	6 (50.00)	

*Fisher’s exact test; *P* ≤ 0.05, statistically significant.

## Discussion

Many studies have reported that the plain radiographs and CT scans were recommended for evaluation missed Monteggia fractures^5^, for using MRI in the preoperative evaluation of missed Monteggia fractures has not reported in the English-language literature. However, most of the intra-articular injuries sustained in missed Monteggia fractures are soft tissue or cartilaginous in nature and may not be well assessed on X-ray and CT. Another disadvantage of X-ray and CT is the inability to fully evaluate a dislocation of the unossified or partially ossified radial head. In addition, MRI offers the additional benefit of no exposure to ionizing radiation to the patient. Preoperative MRI can display the appearance of the radial head, radial notch and the annular ligament and may help to detect the situation of the interosseous membrane^22^. In our study, we observed the appearance of the radial notch, the dislocation of the proximal radioulnar joint, and the rupture of the annular ligament in the axial plane, and both T1- and T2-weighted axial images clearly demonstrated the interosseous membrane^23^ (Fig. 6). In the sagittal plane, we can observe the appearance of the radial head and the tortuosity of the annular ligament. Observation of the dislocation of the humeroradial joint for Bado type III missed Monteggia fracture and the appearance of the radial head and radial notch can be displayed in the coronal images.

In our study, the results showed that the patient’s gender and age had no significant influence on the appearance of radial notch/head and final radiographic results. Additionally, the preoperative appearance of radial notch/head was not related with the surgical procedure but related with the final radiographic results: when the appearance of radial head and radial notch were concave, the rate of radial head subluxation/re-dislocation was lower than that of the flat/domed radial head and radial notch (5.56 vs 81.82%, respectively; P < 0.0001). The interval time was an important factor which related with the appearance of radial notch/head and final radiographic results. That means the longer the interval from injury to operation, the more severe abnormality of the radial head and the radial notch, and the lower the likelihood of a good radiographic result. No patient with the interval time of less than 6 months between the injury and open reduction showed flat/domed radial head and radial notch on preoperative MRI, and these final radiographic and clinical result was satisfied. Lu^24^ mentioned that if the interval time was more than 6 months, the radial head had the tendency to enlarge or overgrow. In our series, there were ten patients for whom the interval between the injury and open reduction was longer than 6 months. Of these ten patients, eight subsequently showed subluxation/re-dislocation of the radial head, which indicated that severe hyperplastic changes in the proximal radius–ulna had a direct correlation with the subluxation/re-dislocation of the radial head, because the overgrowth radial head was incongruency with the capitellum (Fig. 4) and the hyperplasia radial notch hindered the radial head to the correct anatomical position. Therefore, we suggest that severe hyperplasia of the radial head and the radial notch of the ulna should be considered the limiting factors for reconstruction of the radial head.

In this study, our data also suggested that treating a missed Monteggia fracture by open reduction of the radial head and osteotomy of the ulna, with or without annular ligament reconstruction and transcapitellar K-wire, is generally successful: 19 patients still maintained radial head reduced and all of the patients improved clinical scores at the latest follow-up. Most authors advocate open reduction and ulnar osteotomy with or without reconstruction of the annular ligament^15,19,22,25,26^. The advantages of open reduction include direct visualization of the elbow joint to debride and evaluation of the integrity of the annular ligament. For ulna osteotomy, the aim was to keep the radial head in the correct anatomical position by providing intrinsic tension in the interosseous membrane, which was no obvious injury in the MRI findings. Annular ligament may contribute to the stability of radial head reduction, but annular ligament reconstruction (ALR) was not mandatory for all patients, and indications should depend on the intraoperative assessment of radial head stability after correction of ulna malalignment^27^. In our series, 12 patients had concurrent reconstruction of the annular ligament and a humeroradial pin. Of these 12 patients, 6 had the hyperplasia of the radial head and notch in the preoperative MRI and their final radiographic outcomes showed re-dislocation of the radial head and osteoarthritic changes (two patients) or subluxation of the radial head (4 patient), which indicated that the hyperplasia of the radial head and notch tends to increase the instability of the radial head and the annular ligament contributes to the stability of the radial head to a certain extent and the stability of the elbow joint is maintained by the bony structure and joint congruity^28^. The radial heads were stable in other 17 patients during intraoperative evaluation, and 13 patients had the concave radial notch and 16 had the concave radial head in preoperative MRI evaluation, and 13 had the satisfactory final clinical and radiographic outcomes.

The present study has some limitations. First, this retrospective study has its intrinsic drawback. Second, although multivariate analysis with logistic regression is necessary to identify significant risk factors related to outcome, the number of patients in our cohort was insufficiently large to be analyzed in this way.

## Conclusion

Despite such limitations, our data suggest that treating a missed Monteggia fracture by open reduction of the radial head and corrective osteotomy of the ulna is generally successful and preoperative MRI is meaningful for evaluation of the condition of the radial head and the radial notch which is related for the final radiographic result. The interval time from injury to operation exceeds 6 months, the risk of radial notch/head abnormality and radial head subluxation/re-dislocation after operation significantly increase.

## Materials and methods

We retrospectively reviewed the patient database at our hospital between 2014 and 2021. This retrospective study was conducted in accordance with the Declaration of Helsinki and received approval from the Ethics Committee of Hunan Provincial People’s Hospital. Written informed consent was acquired from all patient parents and/or legal guardians. 29 children with a diagnosis of a missed Monteggia fracture and evaluated by the pre-operative X-ray and MRI were treated by open reduction of the radial head and osteotomy of the ulna, with or without annular ligament reconstruction and transcapitellar K-wire. Preoperative MRI was performed for all patients, and T1- and T2-weighted images of the elbow and forearm made in the axial, sagittal, and coronal planes were used to evaluate the pathology of the elbow and injury of the interosseous membrane. The study group included 9 boys and 8 girls with a mean age of 7 years (range 1–13 years) at the time of open reduction. The right elbow was injured in 10 patients, and the left elbow was injured in 19 patients. The median interval between injury and surgical treatment was 11 months (range 1–84 months). The initial Monteggia lesion in 27 patients was classified as Bado type I, and two were Bado type III^29^. The mean period of follow-up was 3 years (range 1–4 years).

Preoperative and postoperative clinical evaluations of the patients included Kim elbow functional scores. The Kim elbow performance score is based on 4 parameters: deformity, pain, range of motion, and function^30^. The preoperative radiographic parameters evaluated included deformity of the radial head, radial notch of the proximal ulna, congruency of the radiocapitellar joint and the situation of the interosseous membranes with X-ray and MRI. According to the MRI presentation, the appearance of the radial notch and radial head could be classified into three types: “concave”, “flat”, and “domed” (Fig. 1).

The postoperative radiographic assessment was based on radial head coverage in the last standard anteroposterior and lateral radiographs of the elbow which was divided into 5 groups^31^, ranging from grade 1, completely concentric, to grade 5, dislocated. Grade 2, 3 and 4 were defined radial head subluxation, grade 5 was defined radial head dislocation.

### Surgical technique

Kocher approach was used to expose the elbow joint^16^. The appearance of the radial head and radial notch were assessed. In all cases, dense fibrous scar tissue which surrounded the radial head was excised because it prevented radial head reduction. A transverse osteotomy of the proximal ulna was carried out to allow reduction of the radial head^15^. Fluoroscopy was used to determine the level (the osteotomy was made approximately 1 cm distal to the coronoid process) and the optimal placement of the plate and screws, avoiding interference of the screws with the growth plate of the proximal ulna. The ulna was overcorrected^21^ and temporarily fixed with a pre-angulated locking compression plate and two screws, one proximal and one distal to the osteotomy. The stability of the radial head was tested in flexion, extension, pronation, and supination under direct visualization^24^. If the radiocapitellar joint was unstable in full pronation and flexion beyond 90° after again adjusting ulna angulation and distraction, the annular ligament was reconstructed using triceps fascia^11^, and the humeroradial joint was pinned with a single 1.6 mm Kirschner wire under direct vision from the posterior aspect of the capitulum with the elbow in full supination and flexion at 90°. This was required in six cases.

Postoperatively, an elbow cast was applied with the elbow flexed to 90° and the forearm in supine position for 6 weeks. The humeroradial pin was also removed at 6 weeks. After removal of the cast, gentle active elbow movement was encouraged.

### Statistical analysis

The SPSS 23.0 statistical software package was used to analyze the data. The measurement data were expressed as the means ± standard deviation ($\tilde{x}$ ± *s*), and t test was used to analyze the data. Numerical data were expressed as the rate (composition ratio), Fisher’s exact test was used to analyze the numerical data. The results with a *P* value < 0.05 indicate that the difference is statistically significant.

## Data Availability

The datasets used and/or analysed during the current study available from the corresponding author on reasonable request.
